# mHealth versus face-to-face: study protocol for a randomized trial to test a gender-focused intervention for young African American women at risk for HIV in North Carolina

**DOI:** 10.1186/s12889-018-5796-8

**Published:** 2018-08-06

**Authors:** Felicia A. Browne, Wendee M. Wechsberg, Paul N. Kizakevich, William A. Zule, Courtney P. Bonner, Ashton N. Madison, Brittni N. Howard, Leslie B. Turner

**Affiliations:** 1 Substance Use, Gender, and Applied Research Program, RTI International, Research Triangle Park, NC USA; 2 Health Policy & Management, Gillings School of Global Public Health, University of North Carolina at Chapel Hill, Chapel Hill, NC USA; 3 Psychiatry and Behavioral Sciences, Duke University School of Medicine, Durham, NC USA; 4 Psychology in the Public Interest, North Carolina State University, Raleigh, NC USA; 5 Research Computing Division, RTI International, Research Triangle Park, NC USA

**Keywords:** HIV testing, Substance use, Alcohol or other drugs (AOD), Key population, Seek and test, Health departments, Young Women’s CoOp

## Abstract

**Background:**

Disparities in the prevalence of HIV persist in the southern United States, and young African American women have a disproportionate burden of HIV as compared with young women of other racial/ethnic backgrounds. As a result, engaging young African American women in the HIV care continuum through HIV testing is imperative. This study is designed to reach this key population at risk for HIV. The study seeks to test the efficacy of two formats of a gender-focused, evidence-based, HIV-risk reduction intervention—the Young Women’s CoOp (YWC)—relative to HIV counseling and testing (HCT) among young African American women between the ages of 18 and 25 who use substances and have not recently been tested for HIV.

**Methods:**

Using a seek-and-test framework, this three-arm cross-over randomized trial is being conducted in three county health departments in North Carolina. Each county is assigned to one of three study arms in each cycle: in-person (face-to-face) YWC, mobile Health (mHealth) YWC, or HCT. At study enrollment, participants complete a risk behavior survey via audio computer-assisted self-interview, and drug, alcohol, and pregnancy screening tests, and are then referred to HIV, gonorrhea, and chlamydia testing through their respective health departments. Participants in either of the YWC arms are asked to return approximately 1 week later to either begin the first of two in-person individual intervention sessions or to pick up the mHealth intervention preloaded on a tablet after a brief introduction to using the app. Participants in all arms are asked to return for a 6-month follow-up and 12-month follow-up, and repeat the survey and biological testing from baseline.

**Discussion:**

The findings from this study will demonstrate which delivery format (mHealth or face-to-face) is efficacious in reducing substance use and sexual risk behaviors. If found to be efficacious, the intervention has potential for wider dissemination and reach.

**Trial registration:**

ClinicalTrials.gov: NCT02965014. Registered November 16, 2016.

## Background

HIV continues to be a significant public health issue in the United States. Disparities in the burden of HIV persist with regard to ethnicity, geographic area, age, and gender. Although African Americans account for 12% of the U.S. population, they are disproportionately affected by HIV [1]. Furthermore, 57% of African Americans living with HIV reside in the southern United States [2, 3], and many are emerging adults. Unfortunately, HIV incidence among young African American women is also striking. For example, African American women aged 20–24 are diagnosed with HIV at almost 12 times the rate of their White counterparts. In North Carolina, young African American women are the most affected group of young women; of the newly diagnosed cases of HIV among young women in 2016, approximately 61% were among African American young women compared with 36% among White young women [4]. These statistics illustrate the gravity and need for interventions to reach young African American women for increased HIV testing and more gender-focused prevention.

Engaging young African American women in HIV testing is essential to improving the health of those who are HIV positive and reducing the risk of transmission to others. Only 45% of African American women reported being tested for HIV in the past year [5]; however, African American women who engage in heterosexual sex account for about 21% of undiagnosed HIV infections [6]. Consequently, many young African American women may be unaware of their HIV-positive status and unknowingly transmit HIV to others. Many young women know it is important to get tested, but they face barriers to testing. Some women may not believe they are at risk for HIV and, as such, do not get tested [7]. Stigma and shame may also influence their decision to get tested [8]. In a study of African American college students in the South, participants cited that barriers to getting tested were lack of knowledge of testing sites, a fear of harming a relationship, and a fear of breaches of confidentiality [9].

The disproportionate burden of HIV among young African American women is largely attributable to high community-level HIV prevalence coupled with condensed sexual networks that increase the risk of infection [10–12]. Given the increased risk of HIV, it is imperative for young African American women to protect themselves during sex. Condom use is the most accessible and inexpensive means of HIV protection to date. However, only 21% of African American women used a condom every time they had sex with their partners in the past year [13], indicating that a significant proportion of women are potentially at risk for HIV.

Several factors have been associated with condomless sex among young African American women. Gender-based violence (GBV) and substance use are associated with sexual risk behavior [14–17]. During their lifetime, about 36% of African American women have experienced sexual violence and 45% of African American women have experienced intimate partner violence (IPV), including sexual and physical violence, and/or stalking by an intimate partner [18]. IPV often creates power inequalities among heterosexual couples—women may not feel empowered to negotiate condom use out of fear for their safety or resistance from their partners—leading to inconsistent condom use [15, 19–22]. Furthermore, African American women may feel that asking their partner(s) to use a condom may imply infidelity [20], or that condoms are unnecessary because they believe they are in an exclusive relationship [23]. Inconsistent condom use is also associated with substance use, because young women who drink alcohol or smoke marijuana are less likely to engage in condom use [14, 16]. More than half (52%) of African American young adults have engaged in illicit drug use, and in a population of students at a historically black college or university (HBCU), 38% and 34% of female students reported binge drinking and marijuana use, respectively [24, 25]. Much like GBV, alcohol or other drug (AOD) use may diminish a woman’s power when engaging in sexual encounters with male partners. Consequently, it is imperative for HIV prevention interventions to address not only sexual risk behaviors but also substance use and violence, and help to promote women’s empowerment [26].

The original Women’s CoOp (WC) is an HIV behavioral risk-reduction intervention that is gender-focused and based in empowerment theory and African American feminism [27]. The WC was developed for African American women who used crack cocaine and tested during a randomized controlled trial in North Carolina. The findings indicated that the women in the intervention arm significantly reduced condomless sex at 6-month follow-up, and had reductions in homelessness and increases in employment at 3-month follow-up, compared with the control and standard-HIV intervention (4-session HIV counseling and health education intervention with HIV testing) group arms [27]. Additionally, women in all groups significantly reduced crack use and sex trading at follow-up. The WC was classified as a best evidence intervention by the Centers for Disease Control and Prevention (CDC) [28], and has been adapted for other populations who use AOD in North Carolina and other settings in the US and internationally [29]. The most recent WC adaptation, the Young Women’s CoOp (YWC), seeks to reduce sexual risk behaviors, AOD use, and violence among young African American women [30, 31]. Among young women aged 16–19 who had dropped out of school or considered dropping out of school, the YWC demonstrated efficacy in reducing condomless sex, and participants in the YWC also had reductions in other outcomes, such as marijuana use and emotional abuse from partners [31]. Of the approximately one-third of participants who opted to test for gonorrhea or chlamydia, 28% tested positive.

Since its inception, the WC and its adaptations have been administered in-person by trained interventionists [29]; however, the advent of mobile health (mHealth) technologies—the delivery of medical and public health information and services through mobile devices—is an innovative approach to HIV prevention that has been used among populations such as female California youth and female African American emerging adults to address barriers to HIV prevention strategies such as condom use and HIV testing [32, 33]. Nevertheless, the literature does not widely address HIV prevention mHealth interventions geared toward sexual risk and HIV testing together with the nexus of substance use and victimization that contribute to HIV risk among young African American women. Given the significant role of AOD use and violence in influencing sexual behavior, creating an mHealth intervention that is woman-focused and addresses the intersections of AOD use, sexual risk, and violence may be essential to alleviating the disparities that persist with HIV.

## Methods

### Aims and objectives

This study seeks to determine the efficacy of a behavioral health intervention, the YWC delivered in two formats, relative to standard HIV counseling and testing (HCT) in reducing risk behaviors such as condomless sex and substance use. More specifically, this study has four aims, as follows: (1) Conduct formative research activities to develop a new, age-appropriate WC intervention (YWC), develop strategies to reach young African American women who use AOD, and develop the YWC interactive mHealth application; (2) test the efficacy of the YWC delivered face-to-face or via mHealth app, relative to HCT; (3) estimate the total costs of delivering the face-to-face YWC and mHealth YWC; and (4) examine participant and health clinic staff perceptions of the face-to-face YWC and mHealth YWC. Below, we discuss the study design, methods, and ethical considerations for the second aim, a randomized trial.

#### Study design

**Fig. 1:**
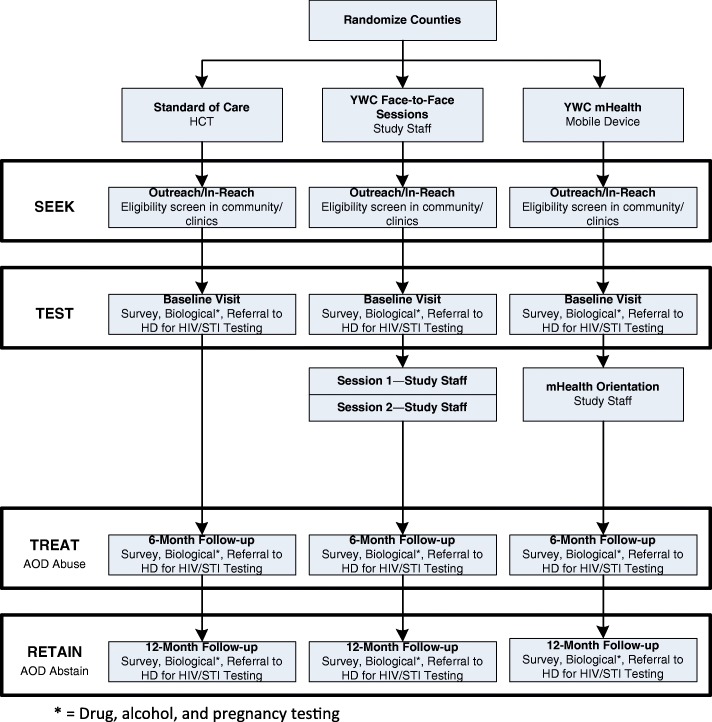
Study design

This study utilizes a randomized three-group, crossover design with two intervention arms and one control arm (shown in Fig. 1). Each county was first randomized to one of three conditions: face-to-face YWC (intervention), mHealth YWC (intervention), or HCT (control)—Cycle 1. Then, controlling for the previous condition, counties were randomized again to determine their study arm for Cycle 2. For Cycle 3, counties will be assigned the remaining study arm. Randomization was conducted via SAS software. We considered each county as a cluster to limit contamination between study conditions, and used a crossover design to account for county differences. This crossover design results in all counties receiving each study arm at some point. Cycle 1 (with the original randomization) occurred from January 2017 to January 2018; Cycle 2 (the first crossover) began in January 2018 and is anticipated to end in October 2018; and Cycle 3 (the second crossover) is anticipated to occur from October 2018 until July 2019.

#### Study setting

In collaboration with county health departments, the Durham County Department of Health, Guilford County Department of Public Health, and Wake County Human Services serve as study sites for the randomized trial. In addition, outreach and recruitment activities are occurring in multiple locations across Durham, Guilford, and Wake Counties. These counties and health departments were chosen because of the high rates of HIV and sexually transmitted infections (STIs) within these counties and for their relative proximity to each other.

#### Eligibility criteria

To be eligible for this trial, individuals must meet the following criteria: (1) be age 18–25; (2) self-identify as Black and/or African American; (3) self-identify as female; (4) have had penetrative sex with a male partner without using a condom within the past 3 months; (5) have used AOD in a greater quantity or for a longer period than they originally intended within the past 30 days; (6) have not tested for HIV within the past 3 months; (7) currently reside in Durham, Wake, or Guilford County for at least the past 6 months; (8) have no intent to move from the area within the next year; (9) have not participated in the previous YWC/Teen CoOp randomized controlled trial or the formative phase of this study; (10) be willing to test for HIV, chlamydia, and gonorrhea through their respective county health departments and sign a release; and (11) be willing to provide locator information for future contact.

During the baseline (i.e., intake) assessment visit, trained project staff first obtain written consent from the potential participant. After the participant signs the consent form, detailed contact information is collected to contact participants for subsequent appointments. Next, participants complete an adapted version of a risk behavior questionnaire (Revised Risk Behavior Assessment) [34] via audio computer-assisted self-interview software, regarding the participant’s AOD use, sexual risk and sexual activity, experiences of victimization and violence, gender roles, communication, and current health status. Participants undergo biological testing for pregnancy and drug use and complete an alcohol breath scan. In addition, participants are referred to the health department for HIV and STI (chlamydia and gonorrhea) testing. These procedures are repeated at the 6-month and 12-month follow-up visits.

#### Study activities by arm

The study comprises three arms, described below. Enrolled participants receive one of the three arms based on the county in which they reside and the cycle in which they enroll.

1. **HCT (control) arm:** Participants in counties assigned to this control arm receive standard HCT and STI testing services from the health department.
2. **Intervention arms:** Participants in counties randomized to the intervention arms complete either the face-to-face YWC or the mHealth YWC, depending on their county assignment at the time of enrollment. The YWC is an age-appropriate, woman-focused intervention that seeks to reduce sexual risk behaviors, reduce AOD use, and develop female empowerment in young African American women. The intervention involves two sessions that include developing personalized action plans and providing participants with condoms and lubricant to practice skills learned during the intervention. The first session of the intervention addresses alcohol, drugs, sexual risks, and relationships, and the second session addresses HIV and condom use, GBV, and conflict resolution. This session also includes a demonstration and rehearsal of both male and female condoms. Throughout the intervention, videos of African American women sharing their stories about these topics are shown. At the end of each session, participants work on personalized action plans to create realistic and achievable risk-reduction and life goals and steps to work toward these goals.

- **Face-to-face YWC:** Participants in the face-to-face arm are asked to return to the health department, or another public location with privacy that is convenient, approximately 1 week after their intake appointment for the first of the two individual intervention sessions with a trained interventionist. The two intervention sessions are targeted to be held 1 week apart.
- **mHealth YWC:** Participants in the mHealth arm are asked to return to the health department, or another public location that is convenient, approximately 1 week after their intake appointment for a brief appointment to pick up their designated Android tablet with preloaded YWC application, and to participate in an introduction to the intervention with a staff member. The staff member shows them how to operate the tablet and navigate the app. Participants create a personal identification number (PIN) for accessing the app, and perform a test upload with the device to ensure that it can connect to Wi-Fi and transmit their encrypted data, to allow for monitoring of their progress. Wi-Fi is not required to use the app, but participants are asked to upload their data periodically, and an upload button in the main menu of the app serves as a reminder. Participants are given the same risk-reduction kit that face-to-face participants are given, which includes male and female condoms and lubricant, as the app contains video demonstrations of how to use them. Participants are also asked to practice during these demonstrations. Participants then take the tablet home to begin the intervention. The mHealth YWC was designed to mirror this in-person delivery via a self-guided mHealth application on a mobile device that allows participants to cover the material at their own pace. The mHealth YWC was developed on the Personal Health Intervention Tool (PHIT) platform [35], which is a generalized toolkit for implementing mHealth applications. Data are stored locally on the mobile device using an encrypted database to await upload to a secure server, where they are available via a password-protected website dashboard. These data are monitored. Participants are asked to return their tablet at their 6-month follow-up appointment.

#### Retention

To assist in participant retention for follow-up appointments, in addition to sessions (if applicable), detailed contact information is collected from participants during their intake appointment. This information includes their address, phone number, social media account(s), places they frequent, locations they would go if they found themselves without a place to stay, whether they give staff permission to visit their address if they cannot be reached, in addition to the contact information of others who are close to them. This detailed information allows for multiple methods of communication to reach participants if their contact information changes. Additionally, to help with participant retention at sessions and follow-up appointments, staff provide transportation or transportation reimbursement, and childcare (if needed).

#### Outcomes

The primary outcomes for this study are self-reported frequency of condomless sex, sexual negotiation (measured by the ability to negotiate condom use and other sexual behavior with a male partner) and substance use. Substance use will also be measured biologically through urine drug screening to assess recent drug use and breathalyzer tests to assess recent alcohol use. Secondary outcome measures include reduced violence and victimization assessed through self-reported experiences of emotional, physical, and sexual abuse. All primary and secondary outcomes will be measured at baseline, 6-month follow-up, and 12-month follow-up.

#### Participant timeline

The first study participant was enrolled in January 2017. Active recruitment is expected to continue until July 2019. We anticipate the duration of each study participant’s involvement to be up to 1 year from the date of first enrollment. The number of contacts a participant is expected to have depends on the study condition to which they are assigned. For the HCT arm, participants have three contacts: intake, 6-month, and 12-month follow-up appointments. Participants in the mHealth arm have one additional contact, an appointment where they pick up their tablets that contain the intervention and learn about the mHealth app (a total of four contacts). Participants in the face-to-face arm have two additional contacts in the form of two intervention sessions (a total of five contacts).

#### Sample size and power

This randomized trial aims to enroll up to 700 young women, with an expected enrollment of 600 based on the power calculation for the study design. We used the Stata command *sampncti* [36], which is based on the equation developed by Chow, Shao, and Wang [37] to calculate the detectable difference in primary outcomes between participants in the control arm and participants in the face-to-face YWC arm. We hypothesize that the participants in the face-to-face YWC arm and participants in the mHealth YWC arm would not have statistically significant different primary outcomes at 6 months; therefore, we calculated the detectable difference based on the control and face-to-face YWC arms. Our parameters were based on a sample size that is feasible given the study design (*n* = 600; 200 participants per arm; an average of 67 participants per condition per county), three counties per arm, intraclass correlation (ICC) = 0.10, and alpha = 0.05, and a two-sided test. All outcome estimates are based on the principal investigator’s (PI’s) previous studies conducted with key populations of young women who use substances in North Carolina [31] and in South Africa [38].

#### Recruitment

To successfully reach and recruit young African American women who use AOD and who have not tested recently for HIV, recruitment strategies such as traditional street-based outreach and marketing efforts are being used. These methods have been successful in other studies with women who use substances in North Carolina [27, 31, 39]. Project staff frequent hotspots—such as bus stops, shopping malls/centers, public housing, and local colleges and universities—and distribute recruitment flyers and cards. These traditional approaches are supplemented with innovative recruitment strategies via digital platforms, such as Facebook, Instagram, and Twitter, and local radio advertisements. In addition, recruitment occurs in each health department—this method of recruitment is referred to as in-reach. With permission, marketing of the study is conducted throughout the health department by project staff. As a supplement to these forms of recruitment, a peer consultant program, which allows for referrals from both study participants and nonparticipants, was implemented to assist in recruitment of potential study participants.

### Modifications to protocol

Several modifications to the study protocol have institutional review board (IRB) approval. Table 1 presents these study modifications. The first three modifications were made prior to participant enrollment.

**Table 1:** Modifications to protocol

Date Approved	Modification
September 2016	Revised eligibility criterion; increased incentive amounts
October 2016	Addition of Certificate of Confidentiality
December 2016	Changed type of drug screening test used; addition of participant stamp card; inclusion of risk-reduction materials for YWC arms
June 2017	Revised eligibility criterion
August 2017	Addition of peer consultant program to assist in recruitment of potential participants for study participation
December 2017	Changed study design from a three-arm parallel group design to a three-arm crossover design; added referral prompts to survey instrument and updated consents to reflect change
January 2018	Changed distressed respondents’ protocol about who to contact

### Data management

All study participants are assigned a unique alphanumeric participant identifier to be recorded on all study-related physical and electronic data sources. Completed data forms are stored in locked file cabinets in rooms located in restricted areas at each health department. Documents that contain identifying information (e.g., name, address, e-mails, phone numbers) are stored in a locked file cabinet separate from study data without these identifiers. Data forms without identifiers are entered and transmitted to RTI through Blaise®, a survey data collection and management system that has programmed data checks; data with identifiers are not entered into Blaise. To ensure the quality of data, staff members are required to review each other’s work. In addition, the project director, study coordinator, and quality assurance coordinator periodically visit each study site to perform quality checks. Hard-copy data forms are destroyed 7 years after study completion.

#### Data analysis

Descriptives, including measures of central tendency (e.g., mean, median), will be used to illustrate the varying characteristics of the study population. The main effect for the primary outcomes will be assessed by analyzing each condition’s change over time between baseline and each follow-up. Differences in rates of change between conditions will be assessed by comparing the slopes from the previous analysis.

#### Missing data

Although attempts will be made to minimize missing data, we anticipate that some data will be missing despite these efforts because of participant attrition and non-response. We will explore patterns of missing data to determine whether the missingness is at random. We will consider data that are Missing at Random (MAR) to be explained by observed covariates. If we believe that missingness is due to unobserved factors (MNAR—Missing Not at Random), then we will use selection models to identify variables to explain it, or pattern mixture modeling. We will examine whether the results of sensitivity analyses are similar with and without missing data adjustments.

#### Data monitoring

A Data and Safety Monitoring Board (DSMB) has been convened to protect the safety of participants and ensure the integrity of collected data. The DSMB meets biannually and is composed of three professionals with expertise in adolescent health and medicine, bioethics and women’s reproductive health, and statistics and research methods, respectively. This board is independent from the researcher and the sponsoring institution for the study. DSMB members discussed whether stopping rules were necessary for this study and they determined that these rules were not necessary.

#### Reporting of adverse events

Because this study presents minimal risk to study participants, study-related adverse events (AEs) or serious adverse events (SAEs) are unlikely. In the rare event that study-related AEs or SAEs occur, staff are directed to immediately notify the project director and PI or designee (a trained clinical psychologist) and submit SAE and incident reports. The PI will report SAEs within 48 h to the National Institute on Drug Abuse (NIDA), RTI’s Office of Research Protection, and the DSMB chair, with a follow-up report submitted within 72 h after initial reporting. The PI will report SAEs to the NIDA Project Officer within 48 h of the event by e-mail. A written follow-up will be sent within 72 h of the event. Additionally, AEs and SAEs will be reported to RTI’s IRB and in reports to the DSMB.

### Ethics and dissemination

The experimental phase of the study received approval from the RTI Committee for the Protection of Human Subjects in August 2016. In addition, research committees of Wake County Human Services and the Durham County Department of Public Health reviewed the study and all related materials with each organization, granting full study approval in November 2016. The Guilford County Department of Public Health Director granted approval in lieu of a formal review by a research committee. All site-based project staff have to complete RTI’s basic human subjects research training module upon hire and before initial contact with any potential study participants. This training will be repeated annually. Furthermore, all staff members responsible for data collection have signed Staff Agreements of Confidentiality to pledge that they will ensure confidentiality of participants and study data. The study findings will be disseminated through our established Community Collaborative Board, program newsletter, journal articles, conference presentations, websites, and targeted dissemination meetings.

## Discussion

HIV among African American women remains disproportionate compared with other racial/ethnic groups of women, especially in the southern United States. It is critical to engage young African American women in the HIV care continuum. This randomized trial seeks to test the efficacy of two formats of the YWC—one is an innovative mHealth delivery and the other is face-to-face delivery—relative to standard HCT. A woman-focused intervention delivered via an mHealth app may be appropriate, especially when adapted for this 18- to 25-year-old population. If this format is found to be efficacious, an mHealth app may succeed in expanding the reach of the HIV risk-reduction intervention, making the YWC more accessible to young women who use mobile devices.

### Trial status

Enrolling.

## Abbreviations

AE: Adverse event
AOD: Alcohol or other drugs
CDC: Centers for Disease Control and Prevention
DSMB: Data and Safety Monitoring Board
GBV: Gender-based violence
HBCU: Historically black college or university
HCT: HIV counseling and testing
HIV: Human immunodeficiency virus
ICC: Intraclass correlation
IPV: Intimate partner violence
IRB: Institutional review board
NIDA: National Institute on Drug Abuse
NIH: National Institutes of Health
PHIT: Personal Health Intervention Tool
PIN: Personal identification number
RTI: RTI International
SAE: Serious adverse event
STI: Sexually transmitted infection
WC: Women’s CoOp
YWC: Young Women’s CoOp
